# Antioxidant, Hypoglycemic, and Neurobehavioral Effects of a Leaf Extract of* Avicennia marina* on Autoimmune Diabetic Mice

**DOI:** 10.1155/2019/1263260

**Published:** 2019-05-21

**Authors:** Mohammad K. Okla, Saud A. Alamri, Abdulrahman A. Alatar, Ahmed K. Hegazy, Abdullah A. Al-Ghamdi, Jamaan S. Ajarem, Mohammad Faisal, Eslam M. Abdel-Salam, Hayssam M. Ali, Mohamed Z. M. Salem, Mostafa A. Abdel-Maksoud

**Affiliations:** ^1^Botany and Microbiology Department, College of Science, King Saud University, P.O. Box 2455, Riyadh 11451, Saudi Arabia; ^2^Department of Botany, Faculty of Science, Cairo University, Cairo, Egypt; ^3^Department of Zoology, Faculty of Science, King Saud University, Riyadh, Saudi Arabia; ^4^Timber Trees Research Department, Sabahia Horticulture Research Station, Horticulture Research Institute, Agriculture Research Center, Alexandria, Egypt; ^5^Forestry and Wood Technology Department, Faculty of Agriculture (EL-Shatby), Alexandria University, Alexandria, Egypt; ^6^Department of Zoology, Faculty of Science, Cairo University, Cairo, Egypt

## Abstract

Diabetes mellitus (DM) is a metabolic disease that can affect the central nervous system and behavioral traits in animals. Streptozotocin-induced diabetes is considered an autoimmune disease. The aim of the current study was to determine whether supplementation with the alcoholic extract of* Avicennia marina* leaves could improve diabetes-associated pathological changes. The animals were divided into four groups: a control group (A), an* A. marina* receiving nondiabetic group (B), a diabetic group (C), and a DM group orally supplemented with* A. marina* alcoholic leaf extract (D). The DM group of animals receiving the alcoholic extract of* A. marina* leaves had reduced blood glucose levels, improved blood picture, and organ functions. This group also showed improvement in locomotory behavior. The results of this study showed that supplementation with the alcoholic extract of* A. marina* leaves reduced oxidative stress and blood sugar levels, protected the liver, and improved the neurobehavioral changes associated with diabetes in mice. Introducing alcoholic leaf extract of* A. marina* to diabetic mice decreased inflammatory cells aggregation, vacuolation, and hemorrhage. Additionally, a positive effect of the alcoholic leaf extract on the histopathological changes was observed in the testicular tissue of treated mice.

## 1. Introduction

Medicinal plants have recently gained much attention from research groups worldwide. The need for new, safer, and effective therapeutic agents represents the main targets for clinical investigators [1]. Owing to the fluctuations in temperature, salinity, and oxygen availability mangrove forests can undergo metabolic pathway adaptations and consequently produce valuable metabolites [2].

*Avicennia marina *is one of the most important mangrove plants that have gained more attention because of its medical importance [3, 4]. Indeed, the study of the medical importance of* A. marina* started early when Bell and Duewel [5] isolated triterpenoids from the bark of* A. marina*. These terpenoids were later identified as lupeol, taraxerol, and betulinic acid [6]. When the antibacterial activity against bacterial specified pathogens was assessed for some mangrove plants, maximum antibacterial activity was observed with the leaf extract of* A. marina* [7]. Additionally,* A. marina *leaf extract has shown antimicrobial activity against some clinical pathogens isolated from urinary tract infections including* Klebsiella pneumoniae *[8]. The antiviral activity of* A. marina* leaf extract was also elucidated [4]. Moreover, the methanolic crude extracts of* A. marina* have inhibited the growth of* Bacillus subtilis, Escherichia coli, Pseudomonas aeruginosa, Staphylococcus aureus, Aspergillus niger, *and* Candida albicans *[9].

Diabetes mellitus (DM) is a worldwide disease with a rapidly growing incidence and severe complications, especially in older individuals. Saudi Arabia ranks as having the second highest incidence of diabetes in the Middle East. It is estimated that around 7 million of the Saudi population are diabetic [10]. “In fact, diabetes has approximately registered a tenfold increase during the last three years in Saudi Arabia” and “DM has been found to be related to high mortality, morbidity, and vascular complications, accompanied by poor general health and lower quality of life” [11]. However, there is a severe lack of studies dealing with herbal therapy for diabetes in Saudi Arabia. Almost all the available data are perspective rather than curative studies [12–14]. Besides, the mangrove ecosystem of the Saudi Arabian Red Sea coast has not yet been investigated enough.

For these reasons, the current study was conducted to investigate the possible therapeutic effects of the alcoholic extract of* A. marina *leaves on streptozotocin (STZ)-induced diabetes in mice.

## 2. Materials and Methods

### 2.1. Animals and Housing

Forty male Swiss Webster (SW) mice were purchased from the animal house (College of Pharmacy, King Saud University). Their average weight was 25-30 gm and they were maintained and monitored in a specific pathogen-free environment. All animal procedures were performed as described elsewhere [15]. The animals had free access to food and water and blood samples were collected at equivalent times relative to feeding.

### 2.2. Diabetes Induction and Experimental Groups

To induce diabetes, groups of animals were intraperitoneally injected with STZ (70 mg·kg^−1^). STZ-injected animals exhibited massive glycosuria and hyperglycemia (200-250 mg·dL^−1^) unlike the control (50-100 mg·dL^−1^) animals within 5 days of STZ administration. Animals were divided into four groups (10 mice/group) as follows: group (A), negative control (administered phosphate buffered saline), neither diabetic nor receiving the extract; group (B), Positive control not diabetic group, receiving the alcoholic extract of* A. marina* leaves; group (C), diabetic; and group (D), diabetic receiving the alcoholic extract of* A. marina* leaves.

### 2.3. Samples Collection and Preparation of Plant Extract

Fresh older leaf samples of* A. marina* were collected from the Jazan district (southwest), Kingdom of Saudi Arabia (KSA). Two hundred grams of dried* A. marina* leaves was chopped into small pieces and soaked in 500 ml of ethanol for 7 days. The colored ethanol solvent was subjected to filtration and kept under a rotary flash evaporator (Buchi, Japan) to obtain the solid extract. The extract was dissolved in 80% ethanol and sterile distilled water was added to prepare a final volume of 100 mg/ml and sterilized by filtration. The animals were administered 2 mg/gm of the extract for four weeks and the dose was calculated according to the average weight of the animals. The percentage of extraction was calculated using the following formula:

(1) Percentage  of  extraction  %=Weight  of  the  extract  gWeight  of  the  dried  plant  material  g×100.

### 2.4. Sample Preparation for Cell Blood Count (CBC) and Histological Analysis

After four weeks of* A. marina* supplementation, mice were prepared for sampling and blood was collected from the heart in heparinized tubes and divided into two parts: one part for the determination of hematological parameters and the other to obtain plasma.

Both liver and testis were removed and cut into small pieces in sterile saline and then fixed in neutral buffered formalin (10%) for histological sections or Tris buffer for biochemical analyses. Sections were cut and stained with hematoxylin and eosin (H/E) and then analyzed under a light microscope (Labomed, Laboamerica, Inc., USA). A pathologist blinded for the experimental regimen performed the pathological evaluation of the H/E stained tissue sections.

### 2.5. Liver and Testis Function Testing

Analysis of plasma samples was performed using commercial kits (Biomerieux, Marcy I'Etoil, France) for alanine aminotransferase (ALT) and creatinine (Creat.) according to the manufacturer's instructions. Absorbance was measured using the Ultrospec 2000 U/V spectrophotometer (Amersham, Pharmacia Biotech, Cambridge, England).

### 2.6. Oxidative Stress Assessment in Hepatic Tissue

Oxidative stress markers were determined in the liver homogenate using commercial kits (Biodiagnostic, Dokki, Giza, Egypt) for nitric oxide (NO), hydrogen peroxide (H_2_O_2_), reduced glutathione (GSH), and malondialdehyde (MDA) according to the manufacturer's instructions.

### 2.7. Antioxidant Activity Assessment in Hepatic Tissue

Antioxidant activity in hepatic tissue was assessed in the liver homogenate using commercial kits (Biodiagnostic, Dokki, Giza, Egypt) for the determination of catalase (CAT) activity according to the manufacturer's instructions.

### 2.8. Locomotory Behavior in the Open-Field Area

After 4 weeks from the start of the experiment, the four animal groups were tested for locomotory behavior using the Ugo Basile 47420-Activity Cage (Italy) that can record the spontaneous coordinate activity in mice and correlate variation of this activity with time.

### 2.9. Statistical Analysis

First, for the normality check of the data, the Anderson-Darling test was applied. The data were normally distributed and are expressed as the mean ± standard error of the mean (SEM). Second, significant differences among groups were analyzed using a one- or two-way analysis of variance followed by Bonferroni's test for multiple comparisons using PRISM (GraphPad Software). Differences were considered statistically significant at* P* < 0.05.

## 3. Results and Discussion

### 3.1. Improved CBC in Diabetic Mice Receiving the Alcoholic Leaf Extract of* A. marina*

CBC is usually used as a biological indicator of the physiological status and pathological consequences of diseases. As described in Tables 1 and 2, aberrant CBC was exhibited by group C as compared with group A. This abnormal CBC was represented as decreased levels of red blood corpuscles (RBCs), white blood corpuscles (WBCs), and hemoglobin (Hb) with a concomitant increase in the levels of hematocrit (HCT), mean corpuscular volume (MCV), mean corpuscular hemoglobin (MCH), mean corpuscular hemoglobin concentration (MCHC), and platelets. Group D showed an improvement in this altered CBC and a restoration to near normal levels was observed. Group B showed a nonsignificant change from the normal values of group A.

### 3.2. Hypoglycemic Effect of the Alcoholic Extract of* A. marina* Leaves on Diabetic Mice

Blood glucose level was determined in all groups of mice. Blood glucose levels were higher in group C (131.4 ± 2.97 mg/dl) (*P* < 0.05) than in group A (77.91 ± 3.76 mg/dl) (Table 3). Oral supplementation with the alcoholic extract of* A. marina* leaves was associated with a significant decrease in the diabetes-associated hyperglycemia. Group D, unlike group A, showed a significant decrease in blood glucose levels (75.5 ± 4.1 mg/dl) (*P* < 0.05). Group B showed blood glucose levels (79.9 ± 3.4 mg/dl) that were not significantly different (*P* > 0.05) from the normal values of the control group (Table 3).

### 3.3. Improved Liver and Testis Functions in Diabetic Mice Receiving the Alcoholic Leaf Extract of* A. marina*

Liver and testis functions were investigated to evaluate the effect of oral supplementation with the alcoholic leaf extract of* A. marina*. As described in Table 4, blood levels of the liver functions indicator, ALT, and the testis functions indicator, Creat., were significantly increased (55.6 ± 4.4 and 3.7 ± 0.1 U/l, respectively) (*P* < 0.05) in group C compared to those in group A. Meanwhile, oral supplementation with the alcoholic leaf extract of* A. marina* had an ameliorating effect on the blood levels of both ALT and Creat. as observed in group D in comparison to that in the control group. Group B showed near normal values of both ALT and Creat.

When investigating the blood level of testosterone hormone in the experimental groups, we found that there was no significant change between the diabetic and the control groups of mice (Table 5). However, when using only the leaf extract, there was a highly significant increase in the testosterone level in the blood samples from the group of mice receiving* Rhizophora* leaf extract [16, 17]. Surprisingly, the concomitant effect of the leaf extract was not significant in the diabetic group of mice compared to that in the control group. This dampening effect on diabetes may be altered when using another part of the plant or another type of solvent.

### 3.4. Hepatoprotective and Antioxidant Effects of the Alcoholic Leaf Extract of* A. marina* in Hepatic Tissue of Diabetic Mice

Oxidative stress is a major pathological sign of many diseases including diabetes. Here, group C, unlike group A, showed increased oxidative stress in hepatic tissues, characterized by increased levels of nitrate, MDA, and H_2_O_2_ with a decrease in the levels of the antioxidant enzymes GSH and CAT (Table 5). Oral supplementation with the alcoholic leaf extract of* A. marina* had a positive effect on diabetes-associated hepatic tissue oxidative stress, whereas it decreased the levels of oxidative stress indicators, nitrate (4.088 ± 0.226 mg/gm), MDA (401.50 ± 33.97 nmol/gm), and H_2_O_2_ (2.620 ± 0.760 mMol/gm) and increased the levels of the antioxidant enzymes GSH (4.389 ± 0.421 *μ*g/g) and CAT (11.872 ± 0.318 nmol/sec/gm). When orally supplemented with the alcoholic leaf extract of* A. marina*, unlike the negative control group mice, the positive control group mice showed a slight increase in the levels of the antioxidant enzymes, GSH and CAT (Table 6).

### 3.5. Ameliorating Effects of the Alcoholic Leaf Extract of* A. marina* on the Diabetes-Associated Behavioral Changes in Diabetic Mice

**Figure 1:**
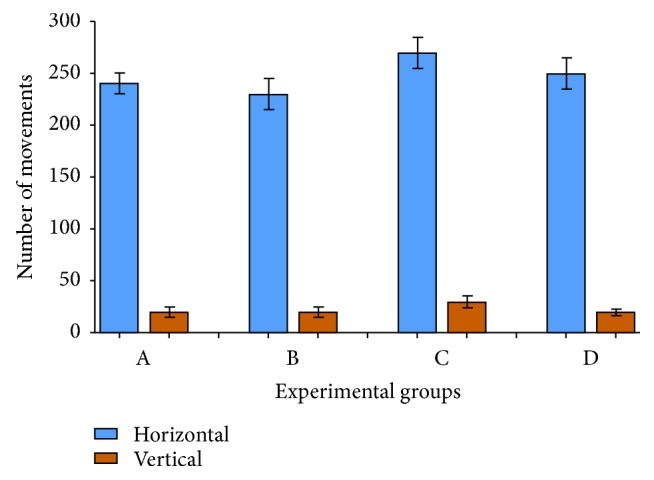
Effect of the alcoholic extract of* Avicennia marina* leaves on the locomotory behavior of the diabetic mice in the activity cage. (A) Negative control group; (B) positive control group; (C) diabetic group; (D) diabetic + AM group (STZ + AM leaf extract). Number of movements per second was plotted for the four experimental groups. AM:* Avicennia marina; *STZ: streptozotocin.

Locomotory behavior in the open-field area was recorded after 4 weeks from the start of the experiment. In the activity cage, the DM group of mice appeared anxious and attained higher scores in the horizontal and vertical activities than the normal and extract-receiving animals (Figure 1). After receiving the alcoholic extract of* A. marina* leaves, group D mice exhibited a mild amelioration in both the horizontal and vertical activities compared to that by group C mice. Surprisingly, group B, receiving only the alcoholic extract of* A. marina* leaves, exhibited the best profile for locomotory behavior.

### 3.6. Effect of the Alcoholic Leaf Extract* A. marina* on the Tissue Sections of Liver

**Figure 2:**
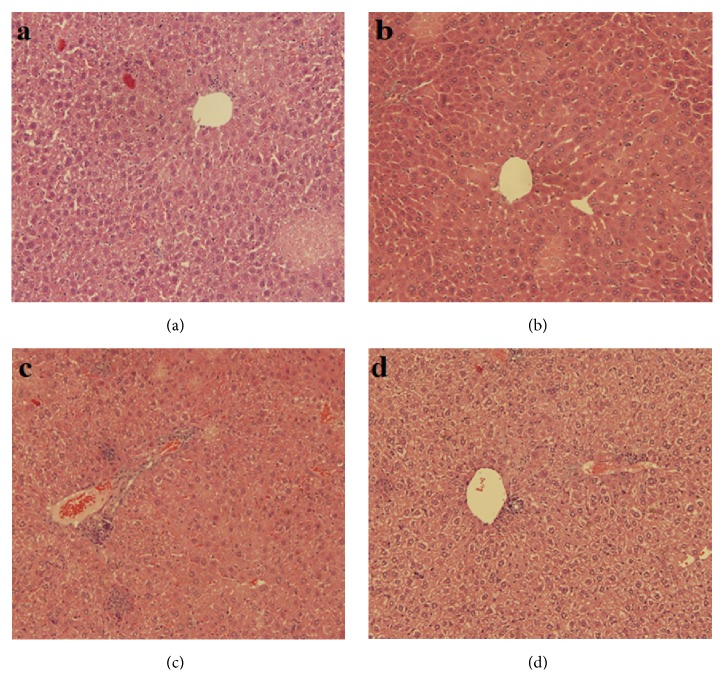
Effect of alcoholic leaf extract of* Avicennia marina* on the tissue sections of liver. (a) Negative control (did not receive any treatment); (b) positive control group receiving the leaf extract; (c) diabetic group (injected with STZ); (d) group receiving STZ + leaf extract. STZ: streptozotocin.

Alcoholic leaf extract of* A. marina* ameliorated the diabetes-associated pathological signs in the liver sections. In the H/E stained liver sections of mice, both group A (Figure 2(a)) and the* A. marina* receiving (Figure 2(b)) groups of mice showed the typical normal structure of hepatic tissue with the strands of hepatocytes arranged around the central vein and normal vascularity. In contrast, tissue sections of group C (Figure 2(c)) exhibited pathological features such as inflammatory cells aggregation, hepatocytic vacuolation, hemorrhage, and edema. Interestingly, the introduction of* A. marina* leaf extract to diabetic mice ameliorated this diabetic-associated tissue pathology as illustrated from the decreased inflammatory cells aggregation, decreased vacuolation, and hemorrhage (Figure 2(d)). These data augment the observation of decreased oxidative stress in liver tissue samples seen in this group of mice.

### 3.7. Effect of the Alcoholic Leaf Extract of* A. marina* on Tissue Sections of the Testis

**Figure 3:**
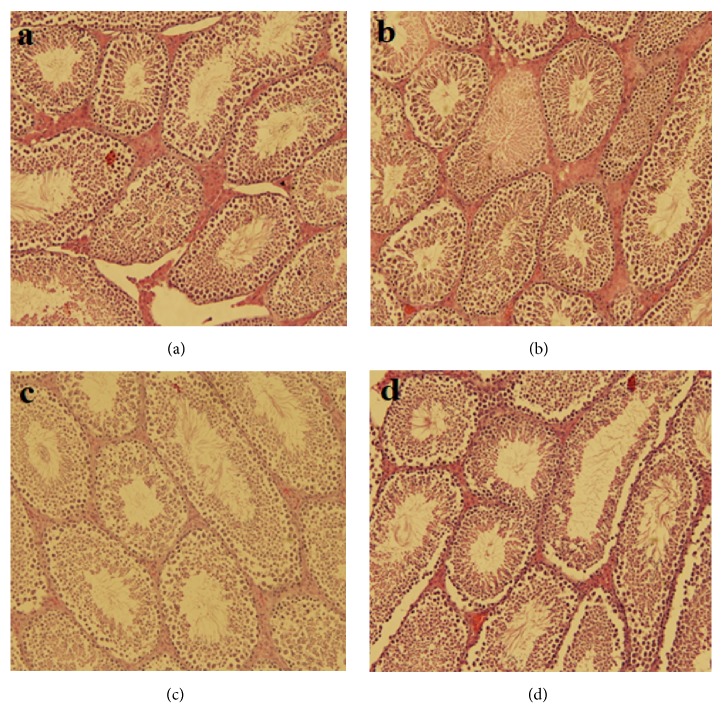
Effect of alcoholic leaf extract of* Avicennia marina* on the tissue sections of testis in mice. (a) Negative control (did not receive any treatment); (b) group receiving the leaf extract; (c) positive control (injected with STZ); (d) group receiving STZ + leaf extract. STZ: streptozotocin.

In the H/E stained testis sections of mice, both the control (Figure 3(a)) and the* A. marina* receiving (Figure 3(b)) groups of mice showed the normal structure of testicular tissue with the characteristic arrangement of seminiferous tubules and the different sperm-forming layers (spermatogonia-primary spermatocyte-secondary spermatocyte). In group C (Figure 3(c)), the situation changed such that the normal structure was disturbed. Vacuolation between tubes, decrease in the sperm number with increase in the number of immature sperms, interstitial edema, and necrosis were the major signs. In the H/E stained testis sections of mice, both of the control and the* A. marina* receiving groups of mice have showed the normal structure of testicular tissue with the characteristic arrangement of seminiferous tubules and the different sperm-forming layers (Spermatogonia- primary spermatocyte-secondary spermatocyte). In the diabetic group of mice, the situation was changed in the way that the normal structure is disturbed. Vacuolation between tubes, decrease in the sperms number with increase in the number of immature sperms, interstitial edema, and necrosis were the major signs. Alcoholic leaf extract showed a positive effect on the histopathological changes in the testicular tissue of treated mice with STZ (Figure 3(d)).

## 4. Discussion

DM is a metabolic disorder that is considered a major health problem and affects millions of people worldwide. The adjunctive use of standardized pharmaceutical-grade nutrients, known as nutraceuticals, has recently gained the increased interest of many research groups [18] and many nutraceuticals are now being used for treating several diseases.* A. marina *is a mangrove plant that could be introduced as a nutraceutical for diabetes.* A. marina* has previously been shown to have an ameliorating effect on experimental diabetic animals. The current study aimed to investigate the possible effects of oral supplementation with ethanolic extracts from* A. marina* on hematological parameters, liver and kidney functions, oxidative stress, and antioxidant parameters in diabetic mice. Our data revealed a disturbed CBC in diabetic mice. Previous studies have reported an altered red cell turn over in diabetic mice [19]. Additionally, in humans, it has also been reported that monocyte counts in the blood of patients with type-1 diabetes being lower than that in patients without diabetes and this was considered as a side effect of diabetes-associated ketosis [20]. In the current study, oral supplementation with the alcoholic extracts from* A. marina* leaves exerted a hypoglycemic effect on the diabetic mice. This is in accordance with previous studies that reported a significant decrease in the blood glucose level in STZ-induced diabetic rats receiving the aqueous- and hydroalcoholic extracts of* A. marina* leaves [21]. The protective effect of* A. marina* against kidney, liver, and cardiac toxicities was recently elucidated [3]. In the present study, we observed an ameliorating effect of the alcoholic extract of* A. marina* leaves on both kidney and liver functions.

The observed amelioration in liver functions of the diabetic mice that were orally supplemented with the alcoholic extracts from* A. marina* leaves may be attributed to the concomitant oxidative stress-lowering effect that was observed in the same group unlike in the control group. It was reported that diabetes is associated with many pathological signs among which is the increased production of free radicals concomitantly with the decreased antioxidant potential [22].

Indeed, persistent hyperglycemia can induce reactive oxygen species (ROS) generation and consequently diabetic-associated pathological complications appear [23, 24]. For example, nitrite generates an oxidant stress and increases NO in EA.hy926 endothelial cells. Nitrite is a breakdown product of NO that in turn is oxidized to nitrate in cells [25] to attenuate intracellular oxidative stress [26]. It has been reported that NO and ROS are associated with several pathophysiological events in hepatic tissue leading to fibrosis and cirrhosis [27]. Our data revealed improved liver functions along with ameliorating effects of diabetes-associated oxidative stress in mice that were orally supplemented with the alcoholic extracts from* A. marina* leaves compared to those in the control group. These findings augment previous reports on the gastroprotective effect of* Avicennia sp.* leaves [28]. At the neurological level, alcoholic leaf extract of* A. marina *has a mild effect on the locomotory activity of SW mice, either diabetic or not. The open-field activity monitoring system used in the current study is a globally accepted method used to measure locomotor and anxiety-like behavior in mice [29] and for monitoring skeletal muscle diseases [16]. Our results indicating no overt behavioral changes in* A. marina* extract-receiving mice are consistent with previous reports [30]. Taken together, our data revealed an ameliorating effect of the alcoholic leaf extract of* A. marina* on diabetes-associated pathology. These effects varied from mild effects to significant ones.

In the current study, the alcoholic leaf extract of* A. marina* showed positive effects on the hepatic tissue pathology of diabetic mice. The ameliorating effect on the histological level was augmented by the biochemical effect of the extract, whereas it exerted oxidative stress-lowering activity in the hepatic tissue as represented by decreased levels of H_2_O_2_, MDA, and NO concomitantly with increased levels of the antioxidants, CAT and GSH. These results are consistent with previous reports [16, 31, 32]. Moreover, the positive effects of the alcoholic leaf extract of* A. marina* on the testicular tissue were numerous. These effects may be partially attributed to the decreased oxidative stress observed in the testicular tissue concomitantly with the increased level of testosterone hormone in blood. The observed effect of the alcoholic leaf extract of* A. marina* on the testicular tissue may be considered as an extension to previous reports [33, 34].

## 5. Conclusion

The alcoholic leaf extract of* A. marina* has antioxidant, hypoglycemic, and neurobehavioral effects on diabetic mice.

## Figures and Tables

**Table 1:** Effect of the alcoholic leaf extract of *Avicennia marina* on the cell blood count (CBC) in mice.

Groups of mice	WBC (×10^9^/L)	RBC (×10^6^/mm^3^)	Hb (g/dl)	HCT (%)	MCV (*μ*m^3^)	MCH (pg)
Group (A)	7.9 ± 0.5	8.50 ± 0.73	15.6 ± 1.8	37.9 ± 3.7	46.8 ± 3.2	17.3 ± 0.4

Group (B)	8.3 ± 0.6	8.6 ± 0.63	15.9 ± 1.99	41.2 ± 5.2	47.3 ± 3.7	17.8 ± 0.8

Group (C)	6.9 ± 0.8	6.84 ± 0.46*∗*	11.8 ± 1.5*∗*	43.3 ± 2.8	52.8 ± 2.2	17.6 ± 0.8

Group (D)	7.4 ± 0.9	7.88 ± 0.09	14.1 ± 2.1	38.6 ± 4.5	48.2 ± 3.1	17.2 ± 0.6

*∗P* < 0.05 for diabetic group of mice vs. negative control; AM: *Avicennia marina;* Hb: hemoglobin; HCT: hematocrit; MCH: mean corpuscular hemoglobin; MCV: mean corpuscular volume; RBC: red blood corpuscles; STZ: streptozotocin; WBC: white blood cells.

**Table 2:** Effect of the alcoholic leaf extract of *Avicennia marina* on the mean corpuscular hemoglobin concentration, platelets, neutrophils, lymphocytes, and monocytes in mice.

Groups of mice	MCHC (%)	Platelets (10^3^/mm^3^)	Neutrophils %	Lymphocytes %	Monocytes %
Group (A)	33.6 ± 1.1	998 ± 33	24 ± 2.1	57 ± 4.4	3 ± 0.54

Group (B)	33.5 ± 0.7	890 ± 70	27 ± 2.2	68 ± 3.1	4 ± 0.66

Group (C)	33.8 ± 1.3	1015 ± 31	29 ± 3.15	78 ± 1.6	4 ± 0.9

Group (D)	32.9 ± 0.9	950 ± 60	22 ± 2.6	52 ± 2.3	4 ± 0.25

MCHC: mean corpuscular hemoglobin concentration.

**Table 3:** Effect of the alcoholic leaf extract of *Avicennia marina* on blood glucose level (mg/dl) in the experimental groups of mice.

Experimental group	Glucose (mg/dl)
Group (A)	(77.91 ± 3.76)

Group (B)	(75.5 ± 3.4)

Group (C)	(131.4 ± 2.97)*∗*

Group (D)	(79.9 ± 4.1)#

*∗P* < 0.05 for diabetic group of mice vs. control; #*P* < 0.05 for diabetic + AM group of mice vs. control. AM: *Avicennia marina*; STZ: streptozotocin.

**Table 4:** Effect of the alcoholic leaf extract of *Avicennia marina* on liver functions in diabetic mice.

Groups of mice	ALT (U/l)	Creatinine (mg/dl)
Group (A)	25 ± 5.1	0.6 ± 0.9

Group (B)	27 ± 6.4	0.66 ± 0.09

Group (C)	55.6 ± 4.4*∗*	3.7 ± 0.1*∗*

Group (D)	30 ± 5.3	0.7 ± 0.05

*∗P* < 0.05 for diabetic group of mice vs. control. ALT: alanine aminotransferase.

**Table 5:** Effect of the alcoholic leaf extract of *Avicennia marina* on blood testosterone level in mice.

Experimental group	Testosterone (ng/ml)
Group (A)	0.020 ± 0.002

Group (B)	0.072 ± 0.005*∗*

Group (C)	0.026 ± 0.001

Group (D)	0.027 ± 0.003

*∗P* < 0.05 for diabetic group of mice vs. control.

**Table 6:** Effect of the alcoholic leaf extract of *Avicennia marina* on oxidative stress parameters in hepatic tissue of mice.

Groups of mice	Nitrate	MDA	H_2_O_2_	GSH	CAT
	(mg/gm)	(nmol/gm)	(mMol/gm)	(*μ*gram/g)	(nmol/sec/gm)
Group (A)	3.148 ± 0.258	363.61 ± 37.21	2.179 ± 0.096	5.793 ± 0.748	9.082 ± 0.990

Group (B)	3.367 ± 0.256	347.15 ± 16.81	2.384 ± 0.088	4.198 ± 0.691	3.537 ± 0.146

Group (C)	5.399 ± 0.196*∗*	597.54 ± 43.16*∗*	5.376 ± 0.226*∗*	2.443 ± 0.424*∗*	3.719 ± 0.071*∗*

Group (D)	4.088 ± 0.226	401.50 ± 33.97	2.620 ± 0.760	4.389 ± 0.421	11.872 ± 0.318

*∗P* < 0.05 for diabetic group of mice vs. control. CAT: catalase; GSH: reduced glutathione; H_2_O_2_: hydrogen peroxide; MDA: malondialdehyde.

## Data Availability

The data used to support the findings of this study are included within the article.
